# Elements for assistance to patients with hematological malignancies to propose care lines: a scoping review

**DOI:** 10.1590/0034-7167-2022-0152

**Published:** 2023-02-06

**Authors:** Mayane Cleisla dos Santos Rocha, Valéria Dantas de Azevedo, Maria de Fátima Lucena dos Santos, Rodolfo Daniel de Almeida Soares, Viviane Euzébia Pereira Santos, Isabelle Campos de Azevedo

**Affiliations:** I Universidade Federal do Rio Grande do Norte. Natal, Rio Grande do Norte, Brazil; II Empresa Brazileira de Serviços Hospitalares. Natal, Rio Grande do Norte, Brazil

**Keywords:** Comprehensive Health Care, Patients, Patient Care Planning, Hematologic Neoplasms, Neoplasms, Atención Integral de Salud, Pacientes, Planificación de Atención al Paciente, Neoplasias Hematológicas, Neoplasias, Assistência Integral à Saúde, Pacientes, Planejamento de Assistência ao Paciente, Neoplasias Hematológicas, Neoplasias

## Abstract

**Objectives:**

to identify the elements for assistance to patients with hematological malignancies to propose a care line.

**Methods:**

this is a scoping review, anchored in the JBI theoretical framework, with searches carried out in April 2021, in eight electronic databases and 10 repositories of theses and dissertations.

**Results:**

the final sample consisted of 93 studies, and the main forms of assistance provided that can support a care line for this public were imaging tests, immunophenotyping, chemotherapy regimens, radiotherapy, infection management, assessment of nutritional status, maintenance of oral function, symptom management and screening for second malignancies.

**Conclusions:**

the elaboration of a care line for onco-hematologic patients is necessary, considering the complexity surrounding the diagnosis and treatment of hematologic malignancies, in addition to the difficulties that are imposed in relation to access and continuity of care in the network.

## RESUMO

**Objetivos:**

identificar os elementos para assistência a pacientes com neoplasias hematológicas para propor uma linha de cuidado.

**Métodos:**

trata-se de uma *scoping review*, ancorada no referencial teórico do JBI, com buscas realizadas em abril de 2021 em oito bases de dados eletrônicas e 10 repositórios de teses e dissertações.

**Resultados:**

a amostra final foi composta por 93 estudos, e as principais formas de assistências prestadas que podem embasar uma linha de cuidado para esse público foram exames de imagem, imunofenotipagem, regimes quimioterápicos, radioterapia, gestão de infecções, avaliação do estado nutricional, manutenção da função oral, gerenciamento de sintomas e rastreio para segundas neoplasias.

**Conclusões:**

a elaboração de uma linha de cuidados para pacientes onco-hematológicos se faz necessária, tendo em vista a complexidade que cerca o diagnóstico e tratamento das neoplasias hematológicas, além das dificuldades que se impõem em relação ao acesso e continuidade do cuidado em rede.

## RESUMEN

**Objetivos:**

identificar los elementos para la asistencia a pacientes con neoplasias hematológicas para proponer una línea de atención.

**Métodos:**

se trata de una revisión de alcance, anclada en el marco teórico del JBI, con búsquedas realizadas en abril de 2021 en ocho bases de datos electrónicas y 10 repositorios de tesis y disertaciones.

**Resultados:**

la muestra final estuvo compuesta por 93 estudios, y las principales formas de asistencia brindadas que pueden sustentar una línea de atención a este público fueron pruebas de imagen, inmunofenotipificación, regímenes de quimioterapia, radioterapia, manejo de infecciones, evaluación del estado nutricional, mantenimiento de la función oral, manejo de síntomas y detección de segundas neoplasias malignas.

**Conclusiones:**

es necesario el desarrollo de una línea de atención al paciente oncohematológico, dada la complejidad que rodea al diagnóstico y tratamiento de las neoplasias hematológicas, además de las dificultades que se imponen en relación al acceso y continuidad de la atención en una red.

## INTRODUCTION

Care lines (CL) refer to proposals for articulating resources and health production practices, based on the most relevant epidemiological needs. This allows defining a care flow through which users must travel through Health Care Networks (RAS - *Redes de Atenção à Saúde*) in order to meet their health needs^(1-2)^.

In view of this, RAS are an organizational model of the health care system, with different technological densities, which are articulated to promote continuous, comprehensive and quality health care. Therefore, they propose to break with the fragmentation of care, by the continuity of health care at all levels of the system, focusing on the management of chronic conditions, concomitantly with acute conditions^(3-4)^.

In this regard, the care network for chronic non-communicable diseases is one of the priority networks, with emphasis on the prevention and control of some health conditions^(5)^, as cancer, in order to reduce the incidence of some types of malignancies and improve the quality of life of users with this disease.

That said, we observed the importance of a CL to organize health care that meets individuals’ needs in the context of onco-hematological diseases, in order to enable the provision of care in a timely manner for promotion, prevention, protection, surveillance, diagnosis, treatment and hematological cancer rehabilitation^(6)^, since they are cancers with a significant incidence in the population^(7)^.

Hodgkin’s Lymphoma (HL), Non-Hodgkin’s Lymphoma (NHL) and Multiple Myeloma (MM) represented, respectively, about 1.4, 7.0 and 2.3 million lives affected by these types of hematological malignancies^(8)^. In addition to this, The incidence rate of Chronic Lymphocytic Leukemia (CLL) and Acute Myeloid Leukemia (AML) has increased considerably in most countries^(9)^ and the incidence of HL cases increased by 38.6% between 1990 and 2017^(10)^.

Such diseases require specialized attention, rapid diagnosis and effective treatment, given their rapid evolution, such as AML, which, although the prognosis has improved in recent decades, only one in four patients survives five years or more^(11)^. In this context, there is a need to systematize the care provided to these patients.

Therefore, it is essential to sum up the elements for the care provided to patients with hematological malignancies to elaborate and propose a CL for such a specific audience, in order to support a safe, quality and assertive care. Moreover, this compilation of knowledge can support clinical decisions and conduct taken, as well as the performance of professionals and the follow-up of patients between health services, therefore, with triggering and improvement of clinical itinerary, in order to ensure the provision of care based on the best available evidence.

Given the above, this study sought to answer the following guiding question: what are the elements for assistance to patients with hematological malignancies that can support the proposition of a CL?

## OBJECTIVES

To identify the elements for assistance to patients with hematological malignancies to propose a CL.

## METHODS

### Ethical aspects

As it was research that used public domain materials and did not involve human beings, it was not necessary to be appreciated by the Research Ethics Committee. However, it is important to highlight that the copyright was respected with correct citation and referencing.

### Study design

This is a scoping review, based on the method proposed by the JBI Reviewer’s Manual and conducted according to the Preferred Reporting Items for Systematic reviews and Meta-Analyses extension for Scoping Reviews (PRISMA-ScR): Checklist recommendations^(12-13)^. The research protocol is registered in the Open Science Framework (https://doi.org/ 10.17605/OSF.IO/VCGPQ).

This type of review aims to investigate scientific evidence, identify and map existing gaps in a given area of study^(14)^. It was developed through five stages: I- definition of research question; II- search for relevant studies; III- selection of studies; IV- data extraction and analysis; V- gathering, synthesis and presentation of results^(15)^.

### Methodological procedures

The first stage included the elaboration of the research question through the PCC strategy (P = Population: onco-hematologic patients; C = Concept: assistance; C = Context: CL).

To complete the second stage, the descriptors that encompassed the largest number of studies related to the research topic were identified, through consultation in the Medical Subject Headings (MeSH), for descriptors in English, and in the Health Science Descriptors (DeCS), for descriptors in Portuguese. This process, added to the employment of the Boolean operator AND, culminated in the development of the following search strategy (with its respective descriptors in Portuguese): (Patients AND Patient Care Planning AND Hematologic Neoplasms).

Initially, as a way to ensure that there are no studies with the same theme registered in the OSF or published, a broad search was carried out on the platform and in databases to identify protocols or reviews with similar themes. From this diagnosis, it was followed with the steps to consolidate the scoping review.

Study selection consisted in identifying texts in the databases and repositories of theses and dissertations by reading title and abstract, for screening the works according to eligibility criteria and reading full text for data extraction. The analyses were performed by peers of reviewers independently and, in case of disagreement, there was discussion for reaching consensus. In cases of doubts and disagreements, the opinion of a third reviewer specialized in the area of the object of study was requested.

For the step of separating, summarizing and reporting the essential elements found, a structured instrument specifically designed for this purpose was used. This tool allowed data synthesis, interpretation and basic numerical analysis of the extent, nature and distribution of studies selected to compose the final sample.

### Data source

The searches were carried out in April 2021 in the following databases and repositories of theses and dissertations: U.S. National Library of Medicine (PubMed), Scopus, Cumulative Index to Nursing and Allied Health Literature (CINAHL), Web of Science, Science Direct, PSYCHINFO, EMBASE, Latin American and Caribbean Literature in Health Sciences (LILACS), The National Library of Australia’s Trobe (Trove), Academic Archive Online (DIVA), Electronic Theses Online Service (EThOS), The Education Resources Information Center (ERIC), Theses Canada, DART-Europe E-Theses Portal, National ETD Portal, Theses and Dissertations from Latin America, CAPES Theses and Dissertations Portal and Portuguese Open Access Scientific Repositories (RCAAP - *Repositórios Científicos de Acesso Aberto de Portugal*).

### Data collection and organization

From the studies included in the final sample, the following variables were extracted: year of publication, country of study, language, methodological design, level of evidence, level of health care, type of hematological cancer, the area of health professionals and CL used for assistance to patients with hematological malignancies. The studies’ level of evidence was classified according to JBI proposal^(16)^, categorized from one (I) to five (V).

Data mapping occurred through the use of a structured instrument, proposed by JBI Reviewers^(13)^, which enabled the identification of essential information from the studies, which made it possible to synthesize and interpret data, in addition to generating the basic numerical analysis of the extent, nature and distribution of the studies incorporated in the review.

### Data analysis

The collected data were organized in Microsoft Excel 2016® spreadsheets, analyzed using simple descriptive statistics and presented in graphs, figures, charts and/or tables, as appropriate, to compose the results of this study.

## RESULTS

**Figure 1:**
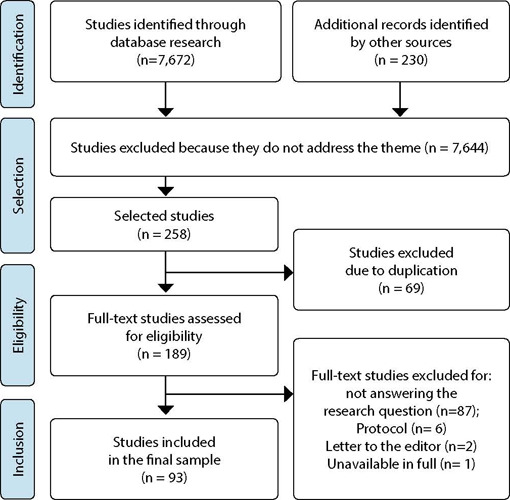
Flowchart of the selection process, adapted according to PRISMA-ScR^(12)^, Natal, Rio Grande do Norte, Brazil, 2021

Searches in the databases resulted in a final sample consisting of 93 studies, based on the selection process described in Figure 1.

Study characterization included in the final sample is described in Chart 1, according to year of publication, country of study, study design, level of evidence, level of health care, type of hematological malignancy and synthesis of CL identified.

**Chart 1:** Study characterization regarding year and country of research development and the elements for assistance to patients with hematological malignancies, Natal, Rio Grande do Norte, Brazil, 2021 (n=93)

Cod*/Year/Country	Elements for assistance to patients with hematological malignancies
A1^†^ / 2012 / Multicenter	**Diagnosis** Imaging tests used in diffuse large B-cell lymphoma, such as computed tomography and positron emission tomography.
A2^†^ / 2017 / United States of America	
A3^†^ / 2016 / Italy	
A4^†^ / 2016 / Colombia	**Diagnosis** Use of gene expression profiling to assess molecular profiling at the time of diagnosis and guide therapeutic decisions, such as next-generation sequencing.
A5^†^ / 2017 / United States of America	
A6^†^ / 2019 / Austria	
A7^†^ / 2018 / United States of America	
A8^†^ / 2019 / Multicenter	
A9^†^ / 2020 / Denmark	
A10^†^ / 2016 / United States of America	
A11^†^ / 2017 / Brazil	**Diagnosis** Clinical techniques, biopsies, complete blood count and differential counts, immunology tests, flow cytometry, radiological tests and genetic technologies such as chromosome analysis and DNA sequencing^‡^.
A12^†^ / 2018 / European countries	
A13^†^ / 2021 / United Kingdom	**Diagnosis** Triple assessment based on clinical examination, imaging and biopsy.
	**Treatment** Surgery is the recommended primary treatment. Mass-forming disease, lymph node involvement, or distant disease may require systemic treatment, with indication for chemotherapy, monoclonal antibody, and/or autologous stem cell transplantation. Radiotherapy should be considered when complete excision is not possible, if surgical margins are positive despite total capsulectomy, or when there is chest wall invasion.
A14^†^ / 2020 / South Korea	**Diagnosis** Use of artificial intelligence-based algorithm for screening patients with suspected hematologic malignancies using cell population data generated by routine complete blood count.
A15^†^ / 2012 / Germany	**Diagnosis** Investigation of central nervous system involvement in the diagnosis of acute myeloid leukemia is considered necessary in children.
	**Diagnosis** Additional tests in diagnosis are medical history, performance status, physical examination, syndromes, comorbidities, biochemistry, clotting tests, serum pregnancy test in women of childbearing potential, hepatitis A, B, C, blood group, chest, 12-lead, electrocardiography, echocardiography.
	**Treatment** Central nervous system treatment is administered to all pediatric patients, including those who have no detectable central nervous system involvement.
	**Treatment** Hematopoietic stem cell transplantation is used as post-remission consolidation therapy.
A16^†^ / 2016 / Multicenter	**Treatment** There is continued indication for allogeneic stem cell transplantation for acute myeloid leukemia, myeloproliferative neoplasia and bone marrow failure, and indication of autologous transplantation in myeloma.
A17^†^ / 2015 / Iran	
A18^†^ / 2017 / United States of America	**Treatment** Allogeneic hematopoietic stem cell transplantation is a treatment option for patients with relapsed and refractory T-cell lymphoma. It should be considered early, in most cases after the first recurrence, since advanced disease and multiple previous therapies predict a higher risk of non-relapse-related mortality and higher recurrence rates.
A19^†^ / 2018 / Germany	
A20^†^ / 2022 / England	**Treatment** Patients under 60 years of age are usually treated with standard induction intensive polychemotherapy, followed by intensive consolidation therapy after remission is achieved. May be associated with hematopoietic stem cell transplantation.
A21^†^ / 2019 / United States of America	
A22^†^ / 2019 / United States of America	
A23^†^ / 2011 / Italy	
A24^†^ / 2008 / Italy	
A25^†^ / 2007 / United States of America	
A26^†^ / 2013 / Austria	
A27^†^ / 2014 / Austria	
A28^†^ / 2016 / China	
A29^†^ / 2016 / United States of America	
A30^†^ / 2017 / South Korea	
A31^†^ / 2018 / Turkey	
A32^†^ / 2017 / South Korea	
A33^†^ / 2018 / Finland	
A34^†^ / 2019 / China	
A35^†^ / 1978 / United States of America	
A36^†^ / 2018 / United States of America	
A37^†^ / 2018 / France	**Treatment** Rescue chemotherapy in case of refractory hematologic malignancies.
A38^†^ / 2019 / United States of America	**Treatment** *Pregnant women* - fetal toxicity occurs from cytostatic therapy groups during the first trimester, while chemotherapy can be safely administered during the second and/or third trimester and the combination of daunorubicin and cytarabine is recommended for induction. All tyrosine kinase inhibitors are teratogenic and are contraindicated during pregnancy.
A39^†^ / 2020 / European countries	
A40^†^ / 2020 / Italy	
A41^†^ / 2017/ Italy	
A42^†^ / 2019 / Taiwan	**Treatment** Brentuximab vedotin, antibody-drug conjugate, is well tolerated and effective in the treatment of Asian patients with relapsed and refractory Hodgkin’s lymphoma. It can strengthen disease control before transplantation and improve post-transplant outcomes, even among heavily pretreated patients.
A43^†^ / 2018 / China	**Treatment** Use of single-mode radiotherapy only in early stages of HL and low-grade NHL. In advanced stage patients, cycles of chemotherapy followed by radiotherapy can be associated. Radiation therapy appeared to improve local control, which suggests that it may be a better choice for therapy when trying to balance treatment efficacy and tolerability.
A44^†^ / 2004 / Australia	
A45^†^ / 2016 / Switzerland	
A46^†^ / 2017 / Italy	
A47^†^ / 2020 / Italy	
A48^†^ / 2020 / Italy	
A49^†^ / 2017 / United States of America	
A50^†^ / 2014 / Netherlands	**Treatment** *Older adults* - patients should be encouraged to make decisions based on accurate information about the risks and benefits of all available treatment options, including investigational clinical trial drugs.
A51^†^ / 2017 / China	**Treatment** Immunotherapy is a safe, viable and well-tolerated treatment for patients with multiple myeloma and may be a promising strategy for relapsed or refractory cases.
A52^†^ / 2015 / China	
A53^†^ / 2019 / United States of America	**Treatment** Immunotherapies are at an investigation stage, but present an attractive means to develop a multimodal therapy with the potential for improved therapeutic efficacy.
A54^†^ / 2013 / Iran	
A55^†^ / 2018 / United States of America	
A56^†^ / 2019 / Canada	
A57^†^ / 2020 / United States of America	
A58^†^ / 2021 / Ireland	
A59^†^ / 2019 / United States of America	**Treatment** *Older adults* - an individualized and personalized approach to administering intensive therapies to older adults with hematologic disorders, with the options of intensive induction chemotherapy, hypomelting agents, allogeneic and autologous hematopoietic cell transplantation.
A60^†^ / 2013 / Italy	
A61^†^ / 2012 / France	**Treatment** *Older adults* - comprehensive geriatric assessment to determine eligibility for treatment, including functional status, comorbidities, cognition, psychological or mental health, social support, nutritional status, and polypharmacy.
A62^†^ / 2007 / Switzerland	
A63^†^ / 2019 / United States of America	
A64^†^ / 2014 / Multicenter	**Support** *Dental* - comprehensive dental and oral assessment to identify and eliminate possible sources of infection, pain and trauma, such as teeth compromised by caries, in addition to pain control, maintenance of oral function, management of oral complications resulting from treatment and conservation of quality of life.
	**Support** *Dental* - guidance on brushing three times a day, using ultra-soft brushes, as well as care with brush hygiene and conservation, in addition to frequent rinsing, especially in situations where hygiene is hampered by oral mucositis.
	**Support** *Dental* - use topical analgesics or anesthetics to relieve pain from oral mucositis.
	**Support** *Dental* - management of oral complications of chemotherapy/stem cell transplantation, such as encouraging the patient to maintain oral intake while suffering from dysgeusia. Maintenance of oral function, such as moistening the mouth with sips and sprays of water or saliva substitutes and mechanical flavor stimulants (chewing gum, sweet and sour candies) and the use of spray or mouthwash.
A65^†^ / 1996 / United States of America	**Support** *Dental* - elimination of potential sources of oral trauma, such as ill-fitting dentures, orthodontic appliances, poor/rough restorations, traumatic dentition and dental calculus.
A66^†^ / 2015 / Brazil	
A67^†^ / 2013 / Japan	**Support** *Dental* - chemotherapy grading tool that induces myelosuppression, to facilitate communication between the medical and dental team and the treatment of odontogenic infection triggered by therapy.
A68^†^ / 2003 / United States of America	**Support** *Multidisciplinary* - screening of symptoms and appropriate intervention, from the implementation of interviews, referrals, coordination and monitoring of nursing. Specialist assessments as part of the initial routine regarding the practice of social service and psychology.
A69^†^ / 2020 / Germany	
A70^†^ / 2019 / United States of America	**Support** Changes in policies for early admission to the Intensive Care Unit for the care of infections in patients with hematopoietic malignancies.
A71^†^ / 2017 / Poland	**Support** *Nutritional -* act prophylactically, based on the assessment of nutritional status, with the aid of various methods (questionnaires to assess the risk of malnutrition, anthropometric measurements, biochemical tests, among others), and introduce nutrient supplementation by enteral nutrition, by providing oral nutritional supplements or parenteral nutrition, in cases of malnutrition and cachexia already installed.
A72^†^ / 2016 / England	**Support** Use low-dose prophylactic platelet transfusions for thrombocytopenic patients due to myelosuppressive chemotherapy or stem cell transplantation.
A73^†^ / 2015 / China	**Support** Protocol implementation to effectively reduce the door-to-antibiotic time to meet the international standard of care in patients with neutropenic sepsis.
A74^†^ / 2006 / South Korea	**Support** Assessment and management of infections associated with neutropenia, with prophylaxis for bacterial and fungal infections due to patients’ compromised immune status.
A75^†^ / 2012 / United States of America	
A76^†^ / 2018 / Italy	
A77^†^ / 2019 / United States of America	**Support** Implementation of yoga therapy protocol in cancer, including body awareness, breathing awareness, adaptive movement, and relaxing imaging practice.
A78^†^ / 2007 / United States of America	**Palliative care** Engage patients in early care planning, which includes the realization of a life will, prognostic discussion, treatment options, and life support treatment preferences.
A79^†^ / 2018 / United States of America	
A80^†^ / 2020 / United States of America	**Palliative care** Manage symptoms, emotional support, attention to psychosocial and spiritual needs, advanced care planning and care coordination.
A81^†^ / 2015 / Finland	
A82^†^ / 2017 / Spain	
A83^†^ / 2015 / United States of America	
A84^†^ / 2019 / Netherlands	**Palliative care** Timely insert eligible patients into care that promotes quality of life, according to individual preferences.
A85^†^ / 2012 / United States of America	
A86^†^ / 2020 / Germany	
A87^†^ / 2014 / Brazil	
A88^†^ / 2019 / United States of America	
A89^†^ / 2019 / Saudi Arabia	**Post-treatment** Screen for secondary malignancies or recurrence of cancer, minimization of risk factors and exposures and early intervention, if detected.
	**Post-treatment** Assess the persistence of treatment-related toxicity and the risks of late effects.
	**Post-treatment** Provide counseling on reproduction and basal fertility assessment, on sperm bank and oocyte bank. In case of sexual dysfunction, perform sex hormone replacement and/or treatment of vaginal dryness.
	**Post-treatment** Screen for psychosocial issues such as depression, anxiety, post-traumatic stress disorder, metabolic syndromes such as diabetes, weight gain or loss, dyslipidemia.
	**Post-treatment** Generate incentive to preventive health, with the practice of healthy life habits, recommendations for regular physical activity, maintenance of optimal body mass index, vaccination, routine screening for fatigue, routine screening and pain assessment, and sleep hygiene education.
A90^†^ / 2019 / China	**Post-treatment** Monitor minimal residual disease, prevention and treatment of recurrence.
A91^†^ / 2000 / Australia	**Post-treatment** Engage patients in support services, such as educational programs, support and volunteers, to support the return to routine at home and provide support to families.
A92^†^ / 2014 / Australia	**Post-treatment** Apply a research tool for unmet needs of survivors in five areas: financial concerns, emotional health, access and continuity of care, information and relationships.
A93^†^ / 2017 / United States of America	**Post-treatment** Propose care models: *1. Advisory Model* The person responsible for care becomes the primary care health professional, who has advanced oncology practice (nurse, oncologist or primary care physician focused on cancer survival). A survival consultation is held with follow-up visits with the formation of survival care plans, summary of late effects, symptom assessment and quality of life. *2. Shared Service Model* The performance of care is shared between the cancer specialist and the primary care physician. In this relationship, the oncologist provides all cancer-related care in the post-treatment phase, while the risk of disease recurrence is higher. During this period, primary care physicians manage non-cancer care, if applicable. When deemed appropriate, the oncologist releases the patient for primary care, including a written summary of treatment in the transition of care and a survival care plan to help formalize communication channels, delegate responsibilities and ensure adequate medical follow-up.

*Cod - Code; ^†^A - article; ^‡^DNA - deoxyribonucleic acid.

There are productions from 1978 to the present day, in which an increase in the last decade stands out, with emphasis on the year 2019 (17; 18.28%). The language of publications was English (93; 100%). Most publications are from the United States of America (USA) (30; 32.26%).

The most developed study design was systematic review (29; 31.18%) and cohort (25; 26.88%). Thus, the level of evidence that stood out was II (29; 31.18%). The assistance provided to people with hematological malignancies stands out due to the levels of health care of high complexity (65; 54.62%) and medium complexity (49; 41.18%). The types of hematological malignancies most addressed in the studies were leukemias (42; 37.5%) and lymphomas (21; 18.75%). Moreover, the most approached type of care was treatment (50; 52.63%), followed by diagnosis (15; 15.79%).

It is noteworthy that some studies portrayed more than one type of care, more than one type of hematological malignancy and the care is applied at different levels of health care, as shown in Chart 1, which justifies the amount of these variables exceeding the amount of the sample (93; 100%).

Article references in the sample of data selected in this research are found in a file attached to the OSF and can be consulted from the access to the platform, through the *link* available in the second paragraph of the method.

## DISCUSSION

The epidemiological relevance of hematological cancer and the interest in improving care for patients affected by this disease are confirmed by the specific care needs, the quantitative of the final sample of this research, the growing number of scientific productions carried out in the last decade and the increase in rates incidence of this type of cancer^(8,9,10,17-18)^.

The most frequent place of studies was the USA and European countries; on the other hand, it is observed that the African continent had no representation, which is possible to correlate with the investment in the technological apparatus and development of scientific productions in these countries^(19-20)^.

Similarly, this relationship may be associated with the magnitude of hematological cancer^(21-22)^ in this group of countries, given that North America has the second highest incidence of NHL cases and Europe ranks third in terms of diagnosis of this disease^(23)^. Moreover, CLL and AML were more commonly diagnosed in countries located in Europe and North America between 1997 and 2017^(9)^.

Furthermore, the most discussed malignancies in the studies were leukemias and lymphomas, in line with the need to provide care to patients, given that these are the types of hematological cancer most distributed among the population^(7,9,20,24,25,26)^.

Regarding the level of health care, the findings of this study reveal that onco-hematological care is concentrated at medium and high complexity levels, since cancer is a type of disease that requires adequate equipment and human resources to provide specialized care^(27-28)^.

However, Primary Health Care (PHC) plays a fundamental role in health promotion, focusing on cancer protection factors, disease screening, early diagnosis, regulation of patients for other levels of care and training of teams, in addition to coordinating and maintaining the care of users with cancer^(29)^.

However, it is observed that there are difficulties in providing care to cancer patients in PHC services, due to the need for qualification of professionals involved in the process of identifying signs and symptoms, referral to early diagnosis and treatment, users’ unequal access to available services and delays in carrying out tests and returning for consultations^(29)^. Thus, it is necessary to improve actions at this level of health care, to minimize the delay in diagnosis, in the provision of care and in the use of resources, in order to guarantee comprehensive care for patients with hematological malignancies.

In this regard, early diagnosis increases success in treatment and chances of cure. Nevertheless, in pediatric leukemia^(30-31)^, where primary prevention is not possible, related to modifiable factors^(32)^, often recommended to adults, such as smoking, alcoholism, sedentary lifestyle and other lifestyle habits, the importance of identifying the various technologies developed for the investigation of the disease is emphasized^(33)^.

Another aspect of remarkable relevance is supportive care^(34)^, since it minimizes complications^(35)^, such as high risk of infections, cachexia, mucositis, depression, loss of social function and other side effects of treatment and impairment caused throughout cancer^(36,37,38,39)^. In addition, it enables the experience of the cancer coping process with better quality of life and better response to malignancy treatment^(40-41)^.

From this perspective, it is still necessary to infer a greater focus of the studies in the curative process, to the detriment of the detection and care directed to post-treatment. With this, there is a growing increase in cancer survivors, from the development and success of anticancer therapies^(42)^.

However, the mortality rate for hematological malignancies is still high^(7,43-44)^. Thus, palliative care aims to improve care during the final life trajectory and should be integrated in a timely manner in standard oncological care. Nevertheless, the implementation of intensive care until the end of life and frequent hospital deaths^(22,45-46)^is still observed.

This is associated with the complex nature of hematological malignancies and their treatment, delays in end-of-life discussions, lack of integration of palliative care services and barriers at home for the patient to experience the end of life at home^(22)^.

On the other hand, increased survival has implied a new demand for post-treatment care that is not yet met by health services, as studies reveal a high prevalence of unmet needs in this population, such as concerns about changes in concentration and memory, fatigue, change in sexual activity, anxiety, depression, and change in body image^(42,47-48)^.

Thus, there is a need for changes in the care of patients with hematological cancer. The determination of a CL that systematizes the therapeutic itinerary of these patients can minimize the existing gaps in the RAS and promote a safe, quality and timely care delivery that can provide a longer survival time^(49)^.

### Study limitations

A limitation of this study is the difficulty in accessing articles that are unavailable in full because they do not meet this criterion, a fact that may have led to the loss of publications that could enrich the findings of this study.

### Contributions to nursing and health

The present study brings relevant contributions to health, especially in the field of onco-hematology, by highlighting the particularities of the elements that can compose a CL to systematize the services and care provided to patients.

It is worth emphasizing the importance of the multidisciplinary team, especially nursing, within this context, as it is the most present professional category throughout the care process. and, thus, with greater opportunities to identify patients’ health-disease needs, outline and make the best decisions and conducts for the implementation of CL, when considering the particularities of each case.

## CONCLUSIONS

In view of the findings, this research achieved the proposed objective and presented the main elements that can support the proposition of a CL for assistance to onco-hematologic patients, when it summarized important aspects about diagnosis, treatment, supportive care, palliative care and post-treatment assistance.

Overall, these patients face several obstacles to achieving specialized care, given the complexity surrounding the diagnosis and treatment of hematological malignancies, as well as difficulties that are imposed in relation to access and continuity of care in the network, which often implies an unfavorable outcome and death.

To this end, this issue needs to be addressed in new research, seeking to raise evidence to base the practice of health professionals and the dynamics of health services, based on the needs presented, associated with the updating and training of health professionals with new knowledge.
